# Intratumoural microbiota: from theory to clinical application

**DOI:** 10.1186/s12964-023-01134-z

**Published:** 2023-06-28

**Authors:** Hao Ji, Zhengting Jiang, Chen Wei, Yichao Ma, Jiahao Zhao, Fei Wang, Bin Zhao, Daorong Wang, Dong Tang

**Affiliations:** 1 Clinical Medical College, Yangzhou University, Yangzhou, 225000 Jiangsu Province China; 2 Department of General Surgery, Institute of General Surgery, Clinical Medical College, Yangzhou University, Northern Jiangsu People’s Hospital, Yangzhou, 225000 China; 3 Clinical Medical College, Dalian Medical University, Dalian, 116044 Liaoning Province China

**Keywords:** Intratumoural bacteria, Tumorigenesis, Anticancer treatment

## Abstract

Cancer is a major cause of high morbidity and mortality worldwide. Several environmental, genetic and lifestyle factors are associated with the development of cancer in humans and result in suboptimal treatment. The human microbiota has been implicated in the pathophysiological process of cancer and has been used as a diagnostic, prognostic and risk assessment tool in cancer management. Notably, both extratumoural and intratumoural microbiota are important components of the tumor microenvironment, subtly influencing tumorigenesis, progression, treatment and prognosis. The potential oncogenic mechanisms of action of the intratumoural microbiota include induction of DNA damage, influence on cell signaling pathways and impairment of immune responses. Some naturally occurring or genetically engineered microorganisms can specifically accumulate and replicate in tumors and then initiate various anti-tumor programs, ultimately promoting the therapeutic effect of tumor microbiota and reducing the toxic and side effects of conventional tumor treatments, which may be conducive to the pursuit of accurate cancer treatment. In this review, we summarise evidence revealing the impact of the intratumoural microbiota on cancer occurrence and progress and potential therapeutic and diagnostic applications, which may be a promising novel strategy to inhibit tumor development and enhance therapeutic efficacy.

Video Abstract

**Supplementary Information:**

The online version contains supplementary material available at 10.1186/s12964-023-01134-z.

## Introduction

Cancer is a complex disease caused by a combination of genetic, environmental and lifestyle factors [1]. The composition and abundance of bacteria in tumors are highly heterogeneous due to host genetics and external environmental factors, which leads to the heterogeneity of tumor morphology and physiological characteristics, and ultimately, difficulty in treatment [2]. The tumor microenvironment (TME) refers to the ecosystem surrounding the tumor, including surrounding blood vessels, immune cells, fibroblasts, bone marrow-derived inflammatory cells, various signal molecules and extracellular matrix (ECM). It plays a crucial role in cancer occurrence and progression. The surrounding host immune cells and cancer cells in the TME contain intracellular microbiota. Tumor microbiota can remodel the TME or stimulate tumor cells to recruit and activate immune cells and related matrix components, thus playing a vital role in the development, treatment and prognosis of cancer [3]. Intratumoural microbiota influences various aspects of tumor occurrence and response to treatment. Cancer development has shifted from a tumor-cell-centered view to the concept of a complex tumor ecosystem [4, 5]. Among the many factors included in the TME, the influence of specific bacteria within the tumor on cancer development and progression has also been studied in recent years [6, 7]. This review explores the effect of intratumoural microbiota on the occurrence and progress of cancer and potential therapeutic and diagnostic applications, to provide a reference for the development of new personalized drugs and improvement of patient prognosis. We also highlight the prospects of intratumoural microbiota as a diagnostic and prognostic marker in cancer patients to improve cancer therapy.

## Intratumoural microbiota and cancer

### Oncogenic role of intratumoural microbiota

**Fig. 1:**
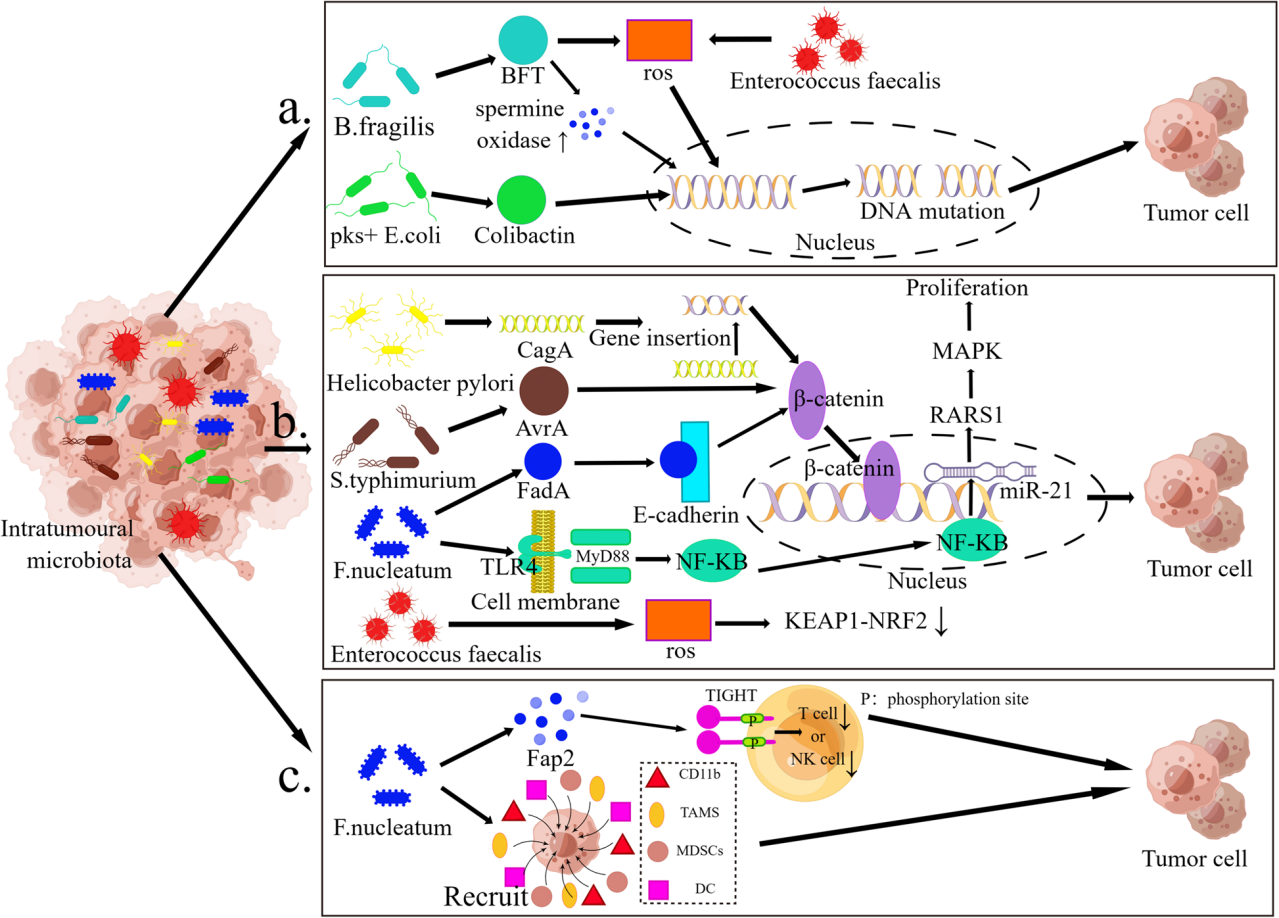
Mechanisms of oncogenesis influenced by intratumoural microbiota. **a** Intratumoural microbiota promotes cancer by damaging DNA. **b** Intratumoural bacteria promotes cancer by affecting cell signaling pathways. **c** Interaction between intratumoural bacteria and the tumor microenvironment

The intracellular microbes found in tumor-like tissues suggest that microbes can not only exist in healthy tissues, but under specific conditions, intratumoural microbiota can use the intrinsic properties of the tumor as an energy source to support microbial proliferation, thus facilitating the survival and rapid proliferation of intratumoural microbiota, and thus achieving colonization of the tumor tissue. However, the biomass of the microbiota in these tumors is relatively low, which makes it difficult to characterise bacteria in tumors [8]. Based on 16S rRNA gene sequencing and characterization, microorganisms in various tumors have been accurately described, and researchers have preliminarily associated specific bacterial species with specific tumor subtypes (Table 1). Table 1 includes 16 types of cancer, including breast cancer, ovarian cancer, prostate cancer, and colorectal cancer, etc. However, the role of intratumoural microbiota in cancer and how they mediate the occurrence and development of cancer remain elusive. Recent studies have shown that intratumoural microbiota may affect the occurrence, progress and prognosis of cancer mainly through various mechanisms such as follows [7, 9, 10]. (1) By causing DNA damage that leads to cell cycle arrest and genomic instability, which increases mutation in tumor tissues occupied by microbiota, ultimately leading to cancer. (2) By modulating signalling molecules that affect cellular signalling pathways, ultimately leading to cancer development. (3) By disrupting the homeostatic equilibrium between intratumoural bacteria and the host immune system, which leads to an immune escape induced by the tumor immune microenvironment and ultimately cancer development (Fig. 1) [5].

**Table 1:** Intratumoural microbiota in different cancer tissues and their role in cancer tumorigenesis, progression and prognosis

Cancer	Status	Intratumoural microbiota	Mechanism	References
Breast cancer	Higher content	*Fusobacterium nucleatum*	1. Causes cancer 2. leads to poor prognosis by suppressing the immune response	[11]
	Reduction	*Anaerococcus, Streptococcus, Propionibacterium*	1. negatively correlated with carcinogenic immune characteristics 2. Positively correlated with T-cell activation-related genes	[12]
Ovarian cancer	Content increasing	*Brucella, Chlamydia, Mycoplasma*	Progressive chromosome loss and translocations cause chromosomal changes and in vitro cell transformation, promoting tumor formation	[13]
Prostate cancer	Higher content	*Pseudomonas, Escherichia, Immunobacterium,* *Propionibacterium spp.*	Induces prostatitis, enhances differentiation of prostate basal cells into ductal cells and promotes tumor formation	[14]
		*Bacillus deformans*	Induces prostatitis and promotes tumor formation	[15]
		*Propionibacterium acnes spp.*	By forming inflammation of the prostate tissue, which in turn leads to the formation of tumors	[16]
	Content increasing	*Staphylococcus*	Induce inflammation of the prostate tissue and promotes tumor formation	[15]
Colorectal cancer	Higher content	*F. nucleic acids*	1. F. nucleatum adhesion molecules bind to cell surface motifs on cancer or immune cells, resulting in downstream oncogenic or immunosuppressive signaling 2. Activation of beta-linked protein signaling; 3. resulting in low CD3 T cell density; 4. causes NK and T cell inactivation	[9] [17]
		*E.coli expressing genomic island polyketide synthase (pks + E.coli)*	pks + E.coli-derived alkylation of DNA by E.coli and production of DNA adducts that lead to DNA damage in colonic epithelial cells and ultimately promote cancer development	[18]
		*Enterotoxin-producing* *Bacteroides fragilis (ETBF)*	ETBF promotes cancer development by recruiting other bacteria and immune cells to the tumor site and promoting IL-17-mediated inflammation	[19]
		*Fusobacterium*	1. Enhances tumor cell adhesion and invasion 2. regulation of host immune response 3. Activates Toll-like receptor 4 pathway	[17]
	Presence	*Bifidobacterium*	Local delivery of bifidobacteria effectively stimulates STING signaling and increases crossover initiation of dendritic cells following anti-CD47 treatment, thereby influencing treatment	[20]
Pancreatic cancer	Higher content	*Enterobacteriaceae,* *Pseudomonas spp.*	Regulates metabolism of chemotherapeutic drugs, leading to chemoresistance and ultimately affecting the efficacy of treatment	[21] [22]
		*Mycobacterium avium*	Influences tumor progression by regulating M1 macrophage/Th1 differentiation that affects CD8 + T cell function	[22]
		*Pseudoxanthomonas,* *Streptomyces, sucrose* *polyspora, Bacillus cereus*	Elevates CD8 T-cell infiltration and activation, affecting prognosis	[22]
		*Malassezia globosa*	Promotes tumorigenesis, tumor growth and gemcitabine resistance through the mannose-binding lectin C3 axis, thereby influencing tumor formation, progression, and prognosis	[23]
Lung Cancer	Higher content	*Acidovorax spp.*	Associated with TP53 mutations	[24]
		*Legionella*	Affects metastasis of cancer	[25]
Esophageal cancer	Higher content	*Lactobacillus fermentum*	Can be used for cancer screening	[26]
		*Campylobacter spp.*	Causes inflammation and affects the prognosis of the tumor	[27]
		*F. nucleic acids*	As a prognostic biomarker	[28]
		*Porphyromonas gingivalis*	1. Promotes immune evasion of tumor cells 2. Inhibits apoptosis of epithelial cells	[29]
		*Fusobacterium nucleatum*	Promote tumor invasion of Treg lymphocytes in a chemokine (especially CCL20) dependent manner, promote aggressive tumor behavior, and affect tumor progression	[30]
	Ecological disorders	*Fusobacterium nucleatum, Streptococcus oligosporus*	Confer chemoresistance to ESCC cells through modulation of autophagy	[30]
Stomach Cancer	Higher content	*Helicobacter pylori*	Enhance tumor formation by promoting p53 degradation and immune escape	[31]
Bladder Cancer	Higher content	*E.coli, butyrate-producing bacteria, oscillating bacilli*	Associates with EMT-related genes, leading to poor prognosis	[32]
Oral cancer	Higher content	*Fusobacterium nucleatum*	Facilitates EMT transition and can be used to predict	[33]
		*Fusobacterium nucleatum, Prevotella*	Causes different types of pulp infections and promotes tumor formation	[33]
		*Streptococcus peptidis*	Enhances anti-tumor immune response and promotes tumor prognosis	[34]
	Ecological disorders	*Mucor (especially Streptococcus), Actinomyces (especially Rhodococcus)*	Promotes cancer and progression	[35]
Cervical cancer	Higher content	*Fusobacterium spp.*	FadA gene overexpression promotes tumor formation	[36]
		*L. crispatus, L. iners*	Promotes tumor formation	[36]
	Ecological disorders	*Lactobacillus lactis,* *Serratia marcescens*	Raising vaginal pH and promoting tumor formation	[37]
Endometrial cancer	Higher content	*Atopobium, Porphyromonas* *Dialister, Peptoniphilus* *Ruminococcus,* *Anaerotruncus* *Anaerostipes, Treponema* *Bacteroides, Arthrospira*	Regulates vaginal pH and promotes tumor formation	[37]
Liver cancer	Higher content	*Helicobacter bifidus*	Induces chronic hepatitis and promotes tumor formation	[38]
Extrahepatic bile duct cancer	Content increasing	*Methanobacterium,* *Fusobacterium, Prevotella, Actinomyces, Neosynovia, H. pylori H*	Increases cagA and vacA gene abundance and promotes tumor formation	[39]
	Reduction	*Helicobacter bilis*	Induces inflammation and promotes tumor formation	[40]
Bile duct cancer	Higher content	*Bifidobacteriaceae,* *Enterobacteriaceae,* *Enterococcaceae*	Metabolic activities can lead to the formation of carcinogens, such as ammonia and bile acids, which promote tumor formation	[41]
Gallbladder cancer	Higher content	*Fusobacterium nucleatum, Escherichia coli,* *Enterobacter spp.*	Promotes the development of gallstones and chronic cholecystitis, which in turn promotes the formation of tumors	[42]

#### Intratumoural microbiota promotes cancer by inducing DNA damage

DNA damage-induced mutation is a critical factor in cancer development and progression. Intratumoural microbiota can cause DNA damage by producing metabolites that cause cell cycle stagnation and genomic instability, ultimately leading to the occurrence of tumors [43]. Studies have shown that various metabolites of intratumoural microbiota can cause cancer development via this pathway. *Escherichia coli* strains of phylogenetic group B2 carry a genomic island known as “pks”, which codes for the production of colibactin, a polyketide-peptide genotoxin. Intratumoural microbiota can induce double-stranded DNA breaks (DSBs) and promotegenomic instability through the secretion of E.coli strains, thereby leading to the development of sporadic colorectal cancer (CRC) [2, 18, 44]. Some Gram-negative bacteria in the γ and ε classes of the *phylum Proteobacteria* can secrete cytolethal distending toxins (CDT) [45]. The catalytic activity of CDTB subtypes has been proved to drive the pathogenic potential of bacteria by directly destroying DNA, thus promoting the occurrence of tumors [46]. It is noteworthy that besides *phylum Proteobacteria*, *Campylobacter jejuni* can also produce CDT, which has DNase activity and causes DSBs [47]. Bacteroides fragilis toxin (BFT) secreted by *Bacteroides Fragilis* degrades E-calmodulin, which causes alterations in signaling pathways that lead to upregulation of spermidine oxidase, which in turn promotes irreversible DNA damage and may eventually lead to carcinogenesis [48]. In CRC cells, Enterotoxigenic Bacteroides fragilis (ETBF) can inhibit exosome-packaged miR-149-3p and subsequently facilitate PHF5A-mediated alternative splicing of KAT2A RNA, ultimately stimulating cell proliferation in CRC [2, 49]. In addition to direct damage to DNA, BFT can also produce high levels of reactive oxygen species (ROS) that can indirectly damage DNA. An increase in the ROS level has long been considered to be related to cancer. Different types of tumor cells have been proven to produce higher levels of ROS than their normal counterparts, thus causing DNA, protein and lipid damage, leading to genomic instability and DNA damage in cancer and promoting genetic instability and tumorigenesis [50]. Notably, in addition to BFT, *Enterococcus faecalis* can produce ROS. Phosphorylated histone H2AX (γH2AX) is a marker for DSBs, and ROS promotes and induces γH2AX lesions of human colon epithelial cells, thus activating the DNA damage pathway, inducing a G2 cell cycle arrest, and promoting chromosome dislocation, leading to aneuploidy and tetraploidy, and finally producing DNA damage in the body [43]. Recent studies demonstrate that adhesive pathogenic bacteria, such as *Enteropathogenic E. coli* and *Enterohemorrhagic E. coli*, can interact with intestinal epithelial cells by leveraging their type 3 secretion system to inject a genetic toxin named UshA. This toxin annihilates DNA in intestinal epithelial cells and results in carcinogenicity [3, 51]. It is noteworthy that certain microorganisms within tumors possess the ability to exert epigenetic effects on DNA repair, and one such microbe is the bifidobacterium. For instance, the restoration of the epigenetically-mediated changes in the human intestinal mucosal immune system can be facilitated by the reduction of histone acetylation and the enhancement of DNA hypermethylation by probiotics such as bifidobacterium. Therefore, to increase the persuasiveness of conclusions drawn from observations of tumorigenesis caused by DNA damage induced by intratumoural microbiota, it is necessary to exclude the epigenetic effects on DNA repair exerted by microbes such as bifidobacterium [52]. These studies suggest that DNA damage is an important mechanism of tumorigenesis mediated by tumor microbes. After comprehending the mechanism of DNA damage, preventive measures can be implemented to delay or avert its occurrence, and prompt intervention and repair can be carried out to preserve genome stability and diminish the probability of tumor development.

#### Intratumoural microbiota activate oncogenic signals

Intratumoural microbiota can also promote tumorigenesis by modulating signaling molecules and influencing cellular signaling pathways. This carcinogenic pathway plays a very important role in the human body. For example, bacteria can affect various signal pathways to cause inflammation or reduce the original protective effect of the signal pathway, leading to cancer. A typical Wingless-related integration site (WNT)/β-catenin signal transduction pathway is involved in several processes such as maintaining tissue homeostasis, regulating cells to affect embryonic development, maintaining self-renewal of stem cells and inducing various tumors [53]. At present, there are numerous studies on *Fusobacterium nucleatum*. Fusobacterium adhesin A (FadA) adhesin produced by *Fusobacterium nucleatum* stimulates the growth of CRC cells, which binds to E-cadherin and activates β-catenin signal transduction, causing inflammation, and leading to cancer [9, 54]. It is noteworthy that Fn activates Wnt/β-catenin signaling. Fn also activates NF-κB and stimulates tumor cell proliferation [54, 55]. Except for *Fusobacterium nucleatum*, FadA expressed by Fn can activate the Wnt/β-catenin pathway. Other substances such as cytotoxin-associated gene A (CagA) protein, avirulence protein A (AvrA), BTF, etc. can also activate the Wnt/β-catenin pathway. For example, the cag pathogenicity island of *Helicobacter pylori* encodes a type IV secretion system that delivers H. pylori-expressed CagA protein into the host cell upon bacterial attachment, leading to aberrant activation of β-catenin, which in turn leads to targeted transcriptional upregulation of genes associated with carcinogenesis, which may ultimately cause gastric cancer [56]. AvrA, secreted by *Salmonella typhimurium* strains that sustain the chronic infection, can cause the development and progression of hepatobiliary cancer by activating epithelial β-catenin signalling [57]. BFT secreted by *B. fragilis*, can stimulate E-calmodulin cleavage and cause β-linked protein activation, which in turn promotes the transcription and translation of the c-Myc proto-oncogene and ultimately induces colon carcinogenesis [58]. In addition, intratumoural bacteria can influence tumor progression by affecting the WNT/β-catenin signalling pathway. For instance, Fn-infected CRC cells can secrete miR-1246/92b-3p/27a-3p-rich exosomes (TEXs) that promote CRC cell migration by targeting GSK3β and activating the Wnt/β-catenin pathway [59], promoting tumor progression. The KEAP1-NRF2 signalling pathway activates antioxidant and detoxifying enzymes and protects humans against chemical carcinogens [60]. The ROS produced by *Enterococcus faecalis* can promote the occurrence and progress of tumors by reducing the protective effect of the KEAP1-NRF2 signal pathway [61]. Jorge et al. showed that exposure to *Fusobacterium nucleatum* resulted in significant upregulation of signaling pathways involved in tumor progression, including extracellular matrix remodeling, metastasis, cell adhesion and migration, as well as EGFR, PDGF, EMT and NF-κB signal pathway is up-regulated [62]. Fn increases cell proliferation by activating Toll-like receptor 4 (TLR4) signalling to myeloid differentiation primary response gene 88 (MyD88) and nuclear factor-κB and then upregulates microRNA-21 (miR-21) expression to directly target the Ras p21 protein activator 1 (RASA1) gene and activate the mitogen-activated protein kinase (MAPK) cascade. Patients with high levels of both Fn DNA and miR-21 in tumor tissues have a poor prognosis [63]. In summary, intratumoural microbiota can contribute to tumorigenesis by modulating cell signalling pathways. In the future, there is a need for further in-depth research into the signal pathways mediated by different intratumoural microbiota in the development of tumors and their relationship with tumor immunotherapy, in order to provide more precise and effective strategies for cancer treatment.

#### Intratumoral microbiota in TME interact with other members

The alteration of tumor immune microenvironment is also thought to be a mechanism of carcinogenesis by intratumoural microbiota [64]. In a healthy state, the interaction between microbiota and the host immune system is in homeostatic equilibrium. The host immune system can tolerate symbiotic microbial communities and respond appropriately to potentially harmful pathogens. However, when the bacterial community is in a state of ecological imbalance, it will lead to a proinflammatory immune response [9], thus forming an immunosuppressive TME that promotes tumor occurrence and progression of tumors [9]. For example, STAT3 plays a crucial role in the initiation and maintenance of immunosuppressive TME during tumorigenesis and progression, while IL-6 is one of the significant activators of STAT3. Upon disturbance of the bacterial community, the IL-6/STAT3 pathway can be activated, resulting in the occurrence of immunosuppression and formation of immunosuppressive TME, ultimately leading to tumor formation [65, 66]. Recent research has indicated that microplastics have the potential to disrupt microbial communities, leading to significant alterations in community composition and structure. These changes can result in elevated expression of key biomarkers, including TLR4, AP-1, and IRF5, which are associated with pronounced inflammatory responses. These inflammatory cascades play a pivotal role in chronic carcinogenic processes and may ultimately lead to tumorigenesis [67, 68]. Intratumoural microbiota can inhibit cytotoxic immune cell infiltration and achieve immune evasion by impairing anti-tumor immunity and blocking its ability to kill tumor cells [12], ultimately affecting tumorigenesis and progression [56]. For example, in a mouse CRC model, interleukin-23 (IL-23) produced by tumor-associated bone marrow cells may be activated by microbial products that penetrate the tumor but not adjacent tissues, thereby triggering tumor-induced inflammation, and eventually driving tumor growth [69]. In addition, intratumoural bacterial communities can impair anti-tumor immune responses by altering antigen presentation and stimulating regulatory T cells (Tregs) and myeloid-derived suppressor cells (MDSCs) [70]. Fibroblast activation protein 2 (Fap2) in Fn directly interacts with T cell immunoglobulin and ITIM domain (TIGIT) expressed by tumor-infiltrating lymphocytes, leading to the inhibition of natural killer (NK) cell cytotoxicity and T cell activity, thus destroying the anti-tumor response and forming an immunosuppressive environment. Fn selectively recruits tumor-infiltrating bone marrow cells (such as dendritic cells (DC), tumor-associated macrophages (TAM), MDSCs and clusters of differentiation molecule 11b (CD11b)) to change the tumor immune microenvironment, thereby promoting tumor progression [55]. These studies explain the role of intratumoural microbiota in tumorigenesis and their importance as potential therapeutic targets for cancer therapy. However, the composition of TME and intratumoural microbiota varies among different organs, indicating potential differences in the underlying mechanisms driving tumorigenesis. How intratumoural microbiota interacts with the TME and whether these interactions can be steered in a positive direction to develop new therapeutic approaches for tumors are fundamental questions that warrant further exploration. Previous studies have provided insights into possible underlying mechanisms to elucidate the role of microbes in tumorigenesis and their importance as potential therapeutic targets for cancer therapy.

### Modulatory effects of intratumoural microbiota in anti-cancer therapy

**Fig. 2:**
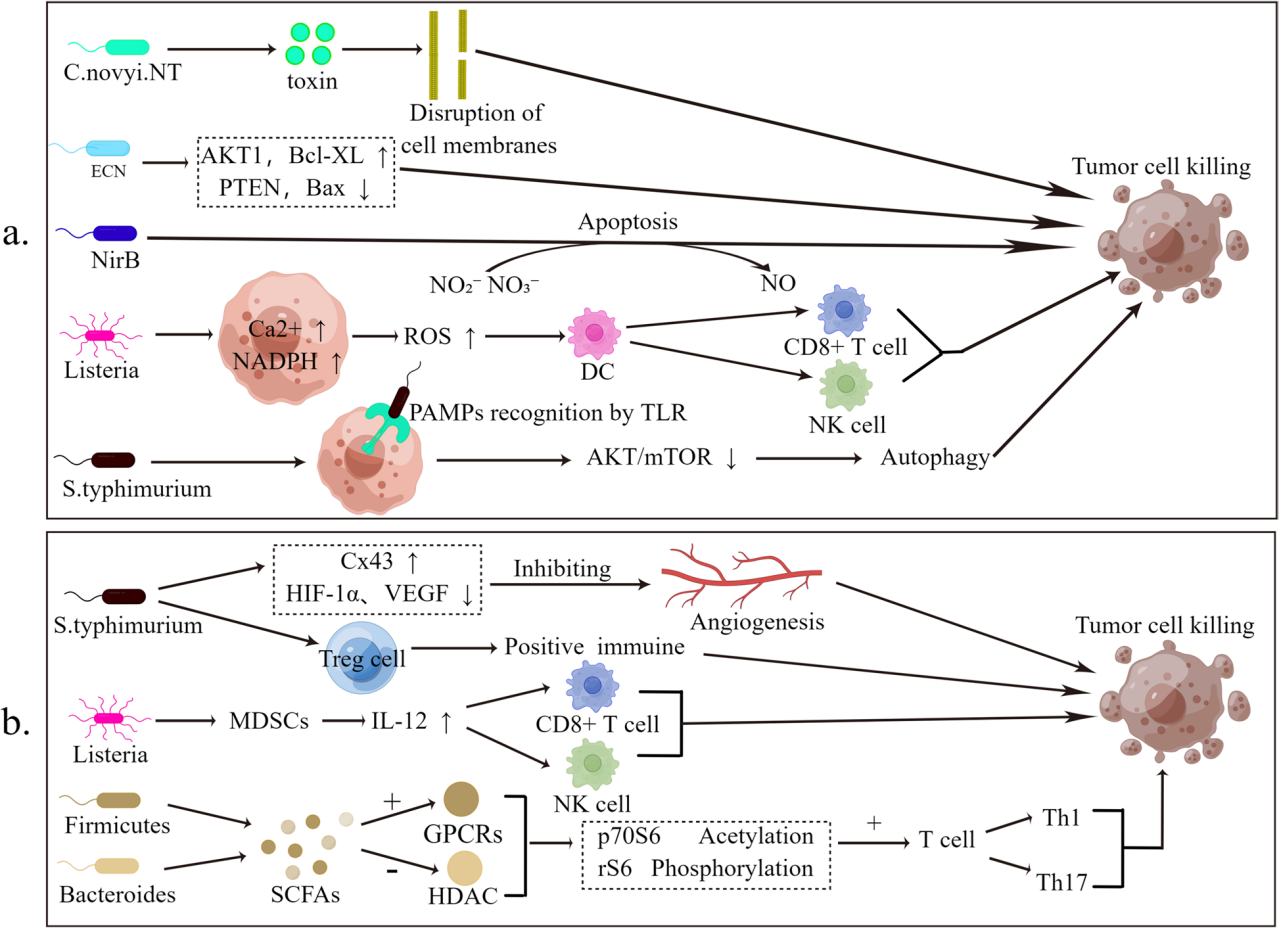
Anti-cancer mechanism of intratumoural microbiota. **a** Intratumoural microbiota can cause apoptosis and autophagy in tumor cells and thus inhibit tumors. **b** Intratumoural microbiota generates an immunosuppressive microenvironment in tumor cells and thus inhibits tumor angiogenesis

Some intratumoural microbiota promote tumorigenesis and progression directly by damaging DNA and influencing signalling pathways, and indirectly by creating a microenvironment conducive to tumorigenesis and progression. However, some intratumoural microbiota can exert tumor suppressive effects and increase the efficacy and reduce treatment-related toxicity of cancer therapy [10]. Intratumoural microbiota that inhibit tumorigenesis and progression can be considered as our friend. While the mechanisms of action of intratumoural microbiota in cancer inhibition are not fully understood, they are currently under further investigation. Some recent studies have suggested that probiotics may exert tumor suppressive effects directly or indirectly mainly by (1) inducing tumor cell apoptosis or autophagy, and directly killing tumor cells and (2) releasing various secretions and metabolites that can enter the circulation to regulate the innate and adaptive immune response. The immunosuppressive microenvironment is transformed into an immunogenic microenvironment, and finally, the tumor inhibition effect is exerted (Fig. 2).

#### Intratumoural microbiota can induce cancer cell death to inhibit tumor progression

Some microbiota in the TME can induce tumor cell death by competing for extracellular nutrients [71], stimulating immune response [72], inducing apoptosis or autophagy signal transduction pathways [73], and releasing bacterial toxins. *E.coli Nissle 1917* (EcN), a probiotic, inhibited the growth of colon cancer by inactivating the expression of AKT1 and anti-apoptotic protein B-cell lymphoma-extra-large (Bcl-xL) which promote the survival of cancer cells and by activating the expression of the tumor suppressor protein phosphatase and tensin homolog (PTEN) and pro-apoptotic protein Bcl-2-associated X (Bax), thereby inducing apoptosis, demonstrating the anti-cancer effect of the probiotic *E.coli Nissle 1917* on CRC cells [74]. *Pediococcus pentosaceus* has been proven to alleviate the development of colon cancer by producing conjugated linoleic acid, which can trigger the apoptosis of colon cancer cells in vivo [75]. *Clostridium Butyricum MIYAIRI 588* (CBM 588) can induce cancer apoptosis and play an anti-tumor role by inducing the release of tumor necrosis factor (TNF)-related apoptosis-inducing ligand (TRAIL) from the intracellular reservoir of polymorphonuclear neutrophils (PMNs) through the TLR2/4 signaling pathway. Matrix-Metalloproteinase-8 (MMP-8) is one of the key molecules in the process of tumor cell apoptosis induction by CBM 588 [76]. In brain cancer cells, the Azurin-like protein Laz produced by *Neisseria Meningitidis*, a bacterium that causes meningitis, is an anti-cancer protein. Laz can induce apoptosis by interacting with tumor suppressor protein p53 [77]. Nitrate and nitrite are reduced by nitrate reductase (NirB) from *Salmonella* [78]. They are further transformed into nitric oxide (NO) in the TME to induce tumor cell apoptosis [79]. In addition to inducing apoptosis, *Salmonella* can also inhibit tumor growth by inducing autophagy. Lee et al. proved that *Salmonella* induced autophagy by downregulation of the AKT/mTOR pathway in a dose and time-dependent manner, which regulated the growth of melanoma in a mouse model. In addition to *Salmonella*, which can inhibit tumor growth by inducing autophagy, short-chain fatty acids (SCFAs, especially acetate) can also inhibit tumor growth by inducing autophagy [80]. Some intratumoural microbiota can directly kill tumor cells. For example, *C. novyi-NT* can secrete various exotoxins, including phospholipases, haemolysins and lipases, which directly kill cancer cells by disrupting cell membranes [81]. Moreover, *Listeria Monocytogenes* (LM) can activate nicotinamide adenine dinucleotide phosphate (NADPH) oxidase and incr ease intracellular Ca2 + levels, both of which lead to the production of high ROS levels, thereby inducing DC activation and maturation. *Listeria* can also infect MDSCs, resulting in an increase in IL-12, which further enhances CD8 T cell and NK cell responses, leading to cancer cell death [82]. Overall, these data suggest that regulating intratumoural microbiota to promote tumor cell apoptosis and autophagy is a feasible treatment method. However, although autophagy is a cell self-protection mechanism, excessive autophagy may cause metabolic stress, degradation of cell components and even cell death. Therefore, in the treatment of tumors, it is necessary to comprehensively consider multiple factors, including the regulation level of autophagy, the type and condition of the tumor, the patient’s physical condition, and the treatment methods used, in order to achieve optimal therapeutic effect (Table 2).

**Table 2:** Intratumoural microbiota that can induce cancer cell death

Intratumoural microbiota	Mechanism	Cancer	References
*E.coli Nissle 1917 (EcN)*	Inducing apoptosis in tumor cells by inhibiting AKT1 and Bcl-xL while activating PTEN and Bax	Colorectal cancer	[74]
*Pediococcus pentosaceus*	Inducing apoptosis in tumor cells by generating conjugated linoleic acid	Prostate Cancer	[75]
*Clostridium Butyricum MIYAIRI 588 (CBM 588)*	Inducing apoptosis in tumor cells through the TLR2/4 signaling pathway	Bladder cancer	[76]
*Neisseria Meningitidis*	Inducing apoptosis by generating Azurin-like protein LAZ	Brain cancer	[77]
*Salmonella*	Inducing hypoxic conditions	Melanoma	[78, 79]
*Salmonella*	Inducing autophagy through downregulation of the AKT/mTOR pathway	Melanoma	[80]
*C. novyi-NT*	Secreting various exotoxins	Colorectal cancer Leiomyosarcoma Liver Cancer	[81]
*Listeria Monocytogenes*	1. Producing high ROS levels 2. Enhancing CD8-T and NK cell responses by increasing IL-12	Colorectal cancer	[82]

#### Intratumoural microbiota produces a tumor-suppressive microenvironment in tumor cells

Solid tumors are difficult to cure because the TME is highly immunosuppressive. For example, Fn stimulates anti-inflammatory myeloid cells in human CRC tissue [55] by activating TIGIT and carcinoembryonic antigen-related cell adhesion molecule 1 (CEACAM1) inhibitory receptors that impair NK and T cell functio [55], creating an immunosuppressive environment conducive to tumor survival and growth [83]. The existence of this immunosuppressive microenvironment limits the efficacy and anti-tumor potential of various immunotherapies. Some intra-tumor microbiota modulate innate and adaptive immune responses by releasing various derivatives that can enter the circulation, transforming the immunosuppressive microenvironment into a tumor suppressive microenvironment or into one that causes immunogenicity [81, 84], and ultimately exerts tumor suppressive effects. SCFAs are produced through the fermentation of dietary fibers, such as butyrate produced by members of the *Firmicutes phylum* and propionate produced by members of the *Bacteroides phylum*. SCFAs can activate G protein-coupled receptors (GPCRs) through different pathways and inhibit histone deacetylases (HDAC) [84], converting the immune system into an anti-inflammatory state to inhibit tumor growth [85]. Studies have shown that SCFAs can increase the acetylation of p70 S6 kinase and phosphorylated rS6 according to different cytokine environments by inhibiting HDAC, thus promoting T cell differentiation into T helper 17 (Th17) and Th1 cells [86]. Meanwhile, a highly attenuated *Listeria Monocytogenes* infects bone MDSCs and changes the immunosuppressive function of MDSCs, transforming MDSCs into an immunostimulatory phenotype that produces IL-12, thereby improving the anti-tumor response of CD8 T cells and NK cells [87]. Notably, because tumors require a dedicated blood supply to maintain oxygenation and other essential nutrients for rapid growth, angiogenesis is the most critical part of tumor transformation from benign to malignant [57, 88]. By inhibiting tumor angiogenesis, it is possible to create a tumor-suppressive microenvironment that is not conducive to tumor growth and thus exerts a tumor-suppressive effect. For example, Salmonella infection can inhibit the expression of vascular endothelial growth factor (VEGF), which stimulates the formation of blood vessels, thus inhibiting tumor angiogenesis. However, there are few examples of Salmonella used to delay the occurrence and development of tumors. Whether the discovery of Salmonella relieving gallbladder cancer can be extended to the whole oncology still needs to be studied and verified. *Salmonella* infection can also induce the up regulation of Cx43 in melanoma model [89], and inhibits angiogenesis through downregulation of HIF-1α and VEGF [90], finally achieve the goal of delaying tumor growth. Therefore, the inhibition of angiogenesis or the destruction of existing blood vessels in tumor tissues by intratumoural bacteria is a potential and feasible therapeutic modality to retard tumor growth. Microplastics have been identified as a potential inducer of colorectal cancer. Recent studies have revealed the capacity of Bifidobacterium to degrade microplastics. Bifidobacterium unique EP structure which has the electrogenic activity and hygrophilic properties for the carbon requirement required for energy provides adhesion on surfaces.It has been discovered that Bifidobacterium can attach to the surface of polypropylene (PP), forming a biofilm that controls the growth of pathogenic bacteria. Additionally, the biofilm reduces the growth of *Escherichia coli O157:H7*, *Listeria monocytogenes*, *Staphylococcus aureus*, and *Salmonellaenterica* on the surface of PP [68, 91]. Additionally, the research indicates that IL-6 is a pro-inflammatory cytokine, and elevated levels of IL-6 are associated with the occurrence and progression of various cancers, including breast cancer, colorectal cancer, gastric cancer, ovarian cancer, and others. Therefore, reducing IL-6 levels may contribute to decreasing the risk of cancer. Bifidobacterium can exert a preventive effect against cancer by lowering IL-6 levels through regulating gut microbiota and immune system mechanisms [91]. A treatment method that is effective in a certain type of tumor may not be practical in other types of tumors. Therefore, understanding how to generalize research results on specific tumors and microbial species to the entire field of oncology, as well as how to convert an immunosuppressive microenvironment to an anti-tumor microenvironment and improve the conversion rate, would have a positive impact on tumor prevention (Table 3).

**Table 3:** Intratumoural microbiota that can produce a tumor-suppressive microenvironment

Intratumoural microbiota	Mechanism	Cancer	References
*Fusobacterium nucleatum*	Stimulation of anti-inflammatory myeloid cells in human CRC tissue by activating TIGIT and CEACAM1 inhibitory receptors, thereby impairing NK and T cell function	Colorectal cancer	[55, 83]
*Firmicutes phylum* *Bacteroides phylum*	Promoting T cell differentiation into Th17 and Th1 cells	Colorectal cancer	[84–86]
*Listeria Monocytogenes*	Transforming MDSCs into an immunostimulatory phenotype that produces IL-12	Breast cancer	[87]
*Salmonella*	Delaying angiogenesis by upregulating Cx43 and downregulating HIF-1α and VEGF	Melanoma	[57, 88, 89]

## The potential clinical value of targeting intratumoural microbiota

Current tumor treatment methods are mainly based on surgery, radiotherapy and chemotherapy [92]. Although these strategies are effective for most tumors, some drawbacks remain.Elucidating oncogenic and anti-cancer mechanisms of intratumoural microbiota may provide a better understanding of the correlation between intratumoural microbiota and cancer development, progression and prognosis, to overcome the drawbacks of conventional cancer therapies and provide key insights into cancer diagnosis and the development of novel therapies.

### Intratumoural microbiota as a potential marker for tumor diagnosis

#### Using intratumoural microbiota for early identification and prevention of cancer

The microbiota community differs significantly between tumor and healthy tissues, and some bacteria are causally linked to cancer development [93]. This suggests the possibility of using intratumoural microbiota as a biomarker for cancer screening [12]. Nejman et al. conducted a comprehensive analysis of tumor microbiota in various tumors and corresponding healthy breast, lung, ovarian, pancreatic, skin, bone and brain tissues and found that each tumor type had a unique microbial composition [5]. For example, sequence analysis of microbes associated with papillary thyroid carcinoma revealed 45, 34 and 33 microbes in classical, follicular variant and tall cell subtypes of papillary thyroid carcinoma, respectively, with differential abundance in tumor and normal tissues [94]. Similarly, the relative abundance of bacteria that can cause DNA damage, such as *Bacillus*, *Enterobacteriaceae* and *Staphylococcus*, increased in breast cancer patients, while the number of lactic acid bacteria, which help to keep the body healthy, decreased [95]. Furthermore, Torres et al. analysed the composition of the oral microbiota in the saliva of pancreatic cancer patients and found a significantly higher proportion of Leptospirillum and porphyria in patients with pancreatic cancer [96]. Notably, familial adenomatous polyposis a precancerous lesion in CRC was found to be a possible indication of the presence of a precancerous inflammatory state by detecting the intratumoural *E.coli*, and thus identifying possible CRC at an early stage [19]. Tjalsma proposed a microbial ecodynamic model of intestinal flora promoting tumor development through DNA damage, the driver-passenger model: driver bacteria with oncogenic properties in the intestine drive epithelial DNA damage and induce CRC production. Subsequently, tumor progression can alter the local microenvironment, affecting the balance of intestinal flora and favoring the proliferation of opportunistic bacteria. Finally, opportunistic bacteria have a growth advantage in the tumor microenvironment, displacing the driver bacteria and leading to further CRC development [97]. Early identification of the occurrence of cancer by detecting intratumoural microbiota and the driver bacteria in the precancerous state may be beneficial to the prevention and early intervention of cancer. These findings suggest that tumor microbiomes may serve as a potential marker for cancer screening and diagnosis. However, to date, most studies regarding intratumoural microbiota have relied on samples obtained through surgery, yet not all cases present with the opportunity for surgery. Therefore, there is an urgent need for more non-invasive methods for detection and diagnosis (Table 4).

**Table 4:** Intratumoural microbiota that can be used for early identification and prevention

Cancer	Intratumoural microbiota	References
Papillary thyroid carcinoma (PTC)		
CPTC	*Rhodococcus fascians D188, Micrococcus luteus, Frankia sp., Anabaena sp. K119, uncultured Gammaproteobacteria bacterium, Trueperella pyogenes, Stenotrophomonas maltophilia K279a*	[94]
FVPTC	*Acinetobacter baumannii AB0057, Micrococcus luteus, Frankia sp., Anabaena sp. K119, uncultured Gammaproteobacteria bacterium, Trueperella pyogenes, Stenotrophomonas maltophilia K279a*	
TCPTC	*Bradyrhizobium sp. BTAi1, Micrococcus luteus, Frankia sp., Anabaena sp. K119, uncultured Gammaproteobacteria bacterium*	
Prostate cancer	*Trueperella pyogenes*	[75]
Cervical cancer	*Bradyrhizobium sp. BTAi1*	[98]
Breast cancer	*Fusobacterium, Atopobium, Gluconacetobacter, Hydrogenophaga, Bacillus, Enterobacteriaceae, Staphylococcus, Comamonadaceae, Bacteroidetes*	[95]
Colorectal cancer	*E.coli*	[19]

#### Using intratumoural microbiota to determine the prognosis of patients.

Intratumoural microbiota can also be used to determine the prognosis of patients with cancer. Studies on esophageal squamous cell carcinoma found that increased levels of intratumoural Fn are associated with advanced tumor stage and poorer survival. The enrichment of Fn in esophageal squamous cell carcinoma tissues may also predict recurrence-free survival [99]. In a different study, researchers performed sensitivity analyses after classifying oral squamous cell carcinoma patients into two groups and found that patients in the Fn-positive group had significantly better outcomes than those in the Fn-negative group. *Fusobacterium nucleatum* also suggested metastatic recurrence: the frequency of metastatic recurrence was more pronounced in cancer patients in the Fn-negative group compared to those in the Fn-positive group [100]. Moreover, the prognosis of primary liver cancer patients was positively correlated with an increased relative abundance of Pseudomonas at the family and genus level, and a significant difference in the predominance of bacterial communities was detected between patients who survived more than 5 years after surgical resection (long-term survivors, LTS) and those who survived less than 5 years after surgery (short-term survivors, STS). LTS tumors exhibited a predominance of *Pseudomonas*, *Thermomonas*, *Paraprevotellaceae*, and other bacteria at the family or genus level. STS cases were dominated by comparable levels of *Enhydrobacter*, *Lachnospiraceae*, and *Deltaprotepbacteria* [101]. Elsewhere, bacterial DNA was extracted from surgically resected pancreatic ductal adenocarcinoma tissues and classified by 16S rRNA gene sequencing and taxonomic analysis. It was found that compared with STS patients, LTS patients had rich and unique microbiomes [102]. Notably, the identification of intratumoural bacteria, including *Sachharopolyspora*, *Pseudoxanthomonas*, and *Streptomyces*, was found to be strongly correlated with long-term postoperative survival in pancreatic cancer patients [102]. Furthermore, recent research has demonstrated that intratumoral microbiomes possess the potential to serve as prognostic indicators for diverse subtypes of thyroid cancer [94]. These studies suggest that the diversity and uniqueness of intratumoural bacteria may have a strong impact on patient survival, confirming a potentially critical link between intratumoural microbiota and clinical prognosis, which may provide a possible method for determining the prognostic status of patients with tumors. It should be noted that further research is needed to demonstrate the accuracy of this method. Although the preliminary results from current research provide initial evidence, more extensive studies are required to validate their efficacy. More detailed and precise data is still necessary to determine whether this method can be used to assess the prognostic status of tumor patients (Table 5).

**Table 5:** Intratumoural microbiota that can be used to predict prognosis

Cancer	Prognosis	Intratumoural microbiota	References
ESCC	Predicting recurrence-free survival (RFS)	*F. nucleatum*	[99, 100]
Primary Liver cancer	Indicating good prognosis (LTS)	*Pseudomonas, Thermomonas, Paraprevotellaceae*	[101]
	Indicating poor prognosis (STS)	*Enhydrobacter, Lachnospiraceae, Deltaprotepbacteria*	
Pancreatic cancer	Indicating good prognosis (LTS)	*Alphaprotebacteria, Sphingobacteria, Flavobacteria*	[102]
	Indicating poor prognosis (STS)	*Clostridia,* *Bacteroidea*	

### Using intratumoural microbiota to improve the efficacy of conventional cancer therapy.

Although traditional chemotherapy and immunotherapy remain the predominant cancer treatment methods, the results are often unsatisfactory, mainly because of the lack of precision and sensitivity [92]. The response of tumor microbes to cancer treatment may be detrimental or beneficial, depending on the treatment method and the potential mechanism of the treatment response (Table 6). However, there is growing evidence that intratumoural bacteria manipulation is a novel and important adjunct to augment conventional anti-cancer therapy, providing more strategies for cancer treatment.

**Table 6:** Impact of intratumoural microbiota on cancer treatment

**Favorable results**						
**Intratumoural microbiota**	**Potential mechanisms**	**Results**	**Tumor type**	**Cells**	**Species**	**References**
*E.coli*	Expression of cytosine deaminase gene of *Escherichia coli* in intratumoural attenuated *Salmonella*	Increased conversion of 5-fluorouracil	Squamous cell and oesophageal adenocarcinoma of the head and neck	Pilot Clinical Trial Organisation	human beings	[103]
HPV	Radiation increases levels of residual double-stranded DNA breaks and G2 blockade in HPV cells	High radiosensitivity of HPV + tumors	Squamous cell carcinoma of the head and neck	hsc4, cal33, sat, ut-5	human beings	[104]
EBV, Herpes virus	(1) Expression of higher levels of drug immune checkpoints (e.g., PD-L1)	Enhanced anti-PD-1/PD-L1 immune checkpoint blockade response in virus-associated cancers	EBV + gastric cancer, herpesvirus-associated Kaposi's sarcoma	Clinical trial tissue with primary human monocytes	human beings	[105]
HIV	(2) Molecular mimicry: recognition of autoantigens initially generated from heterologous antigens			Memory B cells	human beings	[106]
*Salmonella, Listeria*, etc	(3) Direct involvement of innate immunity and TLR-mediated host responses in the tumor microenvironment		Adenocarcinoma of the colon	MC-38	C57BL/6 and BALB/C thymus Nu-/Nu- mice	[107]
*E.coli*	(4) Enhanced acute IFN-g response by outer membrane vesicles derived from *Escherichia coli*		Adenocarcinoma of the colon	CT-26, MC-38	BALB/c and C57BL/6 mice	[108]
Kaposi's sarcoma-associated herpesvirus (KSHV)	(5) Increased expression of pro-inflammatory cytokines (IL-1a, IL1b and IL-6)		Herpesvirus-associated Kaposi's sarcoma	Human mononuclear cells	human beings	[105]
EBV	TCR directly identifies viruses in pericyte therapy	Viruses as targets for immunotherapy	EBV-associated lymphoproliferative disorders after transplantation	Clinical trials	human beings	[109]
**Harmful results**						
**Intratumoural microbiota**	**Potential mechanisms**	**Results**	**Tumor type**	**Cells**	**Species**	**References**
*Gamma Proteobacteria*	Inactivation of the long subtype of cytidine deaminase (CCDL), an enzyme produced by *Gamma Proteobacteria*	Gemcitabine inactivated	Colorectal cancer	MC-26	BALB/c mice	[21]
*F. nucleatum*	Activation of the autophagic pathway through TLR4 and MYD88 immune signalling and miR18 and miR-4802	Resistance to apoptosis by oxaliplatin and fluorouracil	Colorectal cancer	HT-29 HCT116 and SW480	Naked BALB/c mice	[25]
*E.coli*	Reactivation of drugs by B-glucuronidase bacterial enzyme	Increased irinotecan-associated diarrhoea		Proximal colonic cells	BALB/c mice	[110]
	(1) Promotes tolerogenic immune microenvironment by activating specific Toll-like receptors in monocytes	Reduced efficacy of anti-PD-1 immune checkpoint blockade in pancreatic cancer	Ductal adenocarcinoma of the pancreas	KPC cells PAN02 cells	KC, KPC, OT-I, OT-II and C57BL/6WT rats	[111]
*Salmonella*	(2) Bacterial production of metabolites (e.g. SCFA) leading to reduced release of pro-inflammatory chemokines and cytokines			Human monocyte-derived dendritic cells	human beings	[112]
*Campylobacter jejuni*	(3) Bacterial toxins, such as lethal cell swelling toxin (CDT), limit lymphocyte expansion by blocking IL-2 production			70Z/3 and Jurkat3KB5.2 cells	human beings, mouse	[113]
*F. nucleatum*	(4) RecruIntratumoural bacteriaent of tumor-infiltrating myeloid immune cells by *F. nucleatum*		Colorectal cancer		Mice	[55]
*F. nucleatum*	(5) *F. nucleatum* induces alternative immune checkpoints, e.g. TIGIT		Melanoma	Human melanoma cell lines, NK cells, T cells and DCs	human beings	[114]

#### Intratumoural microbiota influences chemotherapy response and toxicity

Although chemotherapy is the mainstay of cancer treatment, it also has significant limitations, including resistance to chemotherapy drugs [115], lack of specificity and accuracy, inability to accurately distinguish between tumors and healthy tissues and side effects that affect the effectiveness of chemotherapy [92], such as intestinal mucositis [116], the most common complication of cancer chemotherapy and chemotherapy-associated diarrhea [117]. Unfortunately, only a few intratumoural microbiota have been observed to alleviate the side effects of chemotherapy drugs. Gui et al. observed that compared with mice receiving cisplatin alone, mice receiving cisplatin combined with *Lactobacillus* showed a better response to treatment. However, the tumor size increased and the survival rate decreased in the lung cancer mouse model receiving cisplatin combined with antibiotics. This indicates that *Lactobacillus* may reduce the toxic and side effects of chemotherapy drugs and improve the efficacy of chemotherapy, thereby improving the survival of lung cancer patients [118]. Chemotherapy-associated diarrhoea is a common adverse effect of CRC treatment. Previous reports found that patients who received *Lactobacillus* during chemotherapy exhibited less abdominal discomfort than those who did not. The frequency of grade 3 or 4 diarrhoea was also reduced and the reduction in chemotherapy dose was less in these subjects, suggesting that *Lactobacillus* had a positive effect on the outcome of chemotherapy [117]. In most cases, intratumoural microbiota is often the cause of resistance to chemotherapeutic agents. For example, *Gamma Proteobacteria* in pancreatic ductal adenocarcinoma (PDA) metabolise the chemotherapeutic drug gemcitabine to its inactive form by expressing the bacterial enzyme cytidine deaminase (CDD), thus inducing gemcitabine resistance and ultimately affecting the efficacy of gemcitabine [21]. There is an urgent need for improved methods based on the negative effects of intratumoural microbiota on chemotherapy, which may help to eliminate the negative effects of intratumoural microbiota. These studies may hint at a need to characterize intratumoural microbiota before treatment, remove intratumoural microbiota that may reduce the efficacy of chemotherapy in tumors by using antibiotics (e.g. *Gamma Proteobacteria* in PDA [21]) or add intratumoural bacteria that may have a reinforcing effect on the efficacy of chemotherapy to the TME, thus improving the efficacy of treatment, which are future directions that should be continuously investigated. It is noteworthy that different types of tumors may require different combinations of microbiota, and further research is needed in this regard. Moreover, the application of antibiotics should be used with caution as it may have adverse effects on the normal microbiota. Therefore, a minimal effective dose and frequency should be considered while ensuring efficacy (Table 7).

**Table 7:** Intratumoural microbiota that can affect chemotherapy response and toxicity

Intratumoural microbiota	Mechanism	Cancer	References
*Lactobacillus*	ABX can partially impair the function of cisplatin by upregulating the expression of VEGFA and downregulating the expression of BAX and CDKN1B	Lung cancer	[118]
*Lactobacillus*	Participating in some cellular protective processes and preventing cytokine induced epithelial cell damage	Colorectal cancer	[117]
*Gamma Proteobacteria*	By expressing the bacterial enzyme cytidine deaminase (CDD), chemotherapeutic drug gemcitabine is metabolized into its inactive form	Pancreatic ductal adenocarcinoma (PDA)	[21]

#### Targeting intratumoural microbiota to treat cancer

Although most intratumoural microbiota are known as tumor promoters, different studies have revealed the therapeutic potential of some intratumoural microbiota. *C. butyricum* is a widely used probiotic with the ability to spontaneously aggregate in colon cancer tissues. Zheng et al. constructed prebiotic-encapsulated *C. butyricum* spores that showed satisfactory anti-tumor effects without any toxicity or side effects [119]. Many other bacterial genera have tumor-targeting and killing activity when administered systemically, suggesting their potential as anti-cancer agents. When specialized anaerobic bacteria such as *Clostridium* are delivered as spores, their colonisation and proliferation are limited to anoxic TME. However, small tumors or metastatic lesions have a high oxygen content, hence facultative anaerobes such as *Salmonella* and *Escherichia* are more suitable [120]. Targeted radionuclide therapy has proven successful in the treatment of several types of cancer, and currently uses radiolabelled small molecules, monoclonal antibodies, peptides and other tumor-targeting vectors [121]. The radioactive particles released by radionuclides physically destroy cancer cells. This therapy has the advantage of not being affected by multidrug resistance mechanisms. However, unsatisfactory results have been reported in trials where monoclonal antibodies have been tried as a targeting vehicle for pancreatic cancer. Thus, new targeting vector options are needed for the successful treatment of pancreatic cancer with targeted radionuclide therapy. In this regard, *Listeria Monocytogenes* offers an attractive system for the delivery of radionuclides to the microenvironment of metastatic and primary tumors. In a mouse model of pancreatic cancer, live-attenuated *Listeria Monocytogenes* in combination with radionuclide 188 rhenium (188Re), *Listeria Monocytogenes* is delivered to TMEs by infecting MDSCs to avoid immune clearance. Then *Listeria Monocytogenes* overgrows to provide radioactivity for metastasis of pancreatic cancer, thus killing tumor cells without serious side effects [122]. This approach could pave way for a unique era in the treatment of metastatic pancreatic cancer. Despite facing various challenges, including the identification of suitable intratumoural microbiota as therapeutic targets, penetration inside tumors, and the assurance of safety and efficacy of treatment, current research in this field demonstrates considerable potential for further advancement. It may be attractive for the clinical development of targeted therapies and could be applied to a wider range of cancer treatments (Table 8).

**Table 8:** Intratumoural microbiota that can be targeted

Intratumoural microbiota	Characteristics	Results	References
*C.butyricum*	Capable of spontaneous aggregation within Colorectal cancer tissue	Promising anti-tumor efficacy coupled with relatively low toxicity and adverse effects	[119]
*Clostridium*	Colonization and proliferation are limited to anoxic TME	Promising anti-tumor efficacy	[120]
*Salmonella, Escherichia*	In the TME with relatively high oxygen content, colonization and proliferation were observed	Promising anti-tumor efficacy	[120]
*Listeria Monocytogenes*	Combined with radionuclide 188 rhenium (188Re) to provide radioactivity to pancreatic cancer metastasis	Good anti-tumor effect without serious side effects	[122]

#### Synergy between intratumoural microbiota and immunotherapy

Immunotherapy has played a vital role in the treatment of tumors; however, conventional immunotherapy has significant limitations, such as the low sensitivity of many patients to immunotherapy and resistance over time. Notably, the specific mechanisms underlying immunotherapy resistance and sensitivity remain unclear [123]. Accumulating studies have shown that intratumoural microbiota can be combined with immunotherapy, offering renewed hope for increased efficacy of immunotherapy [20, 22]. Shi et al. observed that *Bifidobacterium* could accumulate in the TME. Systemic administration of *Bifidobacterium* can lead to its accumulation in the tumor, converting non-responder mice into responders to anti-CD47 immunotherapy in a stimulator of interferon genes (STING) and interferon-dependent manner, ultimately promoting the efficacy of anti-CD47 immunotherapy [20]. A similar effect has been found in immunotherapy of pancreatic cancer. Intratumoural microbiota reprograms the TME of PDA by modulating the PDA microbiome, which reduces MDSCs and increases M1 macrophage differentiation, resulting in increased Th1 differentiation of CD4 + T cells and CD8 + T cell activation, ultimately enhancing the efficacy of immunotherapy [22]. After studying the intratumoural microbiota of melanoma patients, it was found that *Clostridium* was more abundant in patients who responded to immunotherapy, while *Gardnerella vaginalis* was more abundant in patients who did not respond to immunotherapy [5], which indicates that different tumor microbiomes play different important roles in immunotherapy. It may be possible to enrich the tumor microbiome of patients who respond to immunotherapy to achieve the desired therapeutic effects in patients who do not respond to immunotherapy. Thus, the use of intratumoural microbiota as an adjunct to conventional immunotherapy is a viable approach that provides new ideas in cancer treatment. Several studies have demonstrated the efficacy of intratumoural microbiota-assisted immunotherapy in solid tumors. For example, engineered bacteria-mediated antigen delivery is a promising strategy for cancer therapy. After intravenous injection, antigen-secreting engineered bacteria can colonise tumor tissues and induce infiltration of immune cells. Then the antigen secreted by the colonised bacteria leads to the activation of T cells within the tumor to attack the tumor cells. This strategy can effectively shape the anti-tumor immune response of the host and significantly inhibit tumor growth. *E.coli TOP10* is such an example. Because of the induction of tumor-specific CD4 + and CD8 + T cells, significant tumor reduction can be induced in colon cancer mouse models. CD8 + T cells are the only effector responsible for tumor clearance in the induction phase, while CD8 + and CD4 + T cells are involved in the memory phase [124]. Although numerous studies have indicated a correlation between intratumoural microbiota and the efficacy of immunotherapy, the underlying mechanisms remain incompletely understood. These findings may pave the way for the optimisation of this promising therapy. However, further studies are needed to discover intratumoural bacteria that has an enhanced effect on immunotherapy and better understand the specific mechanisms through which intratumoural bacteria influences immunotherapy (Table 9).

**Table 9:** Intratumoural microbiota that can cooperate with immunotherapy

Intratumoural microbiota	Mechanism	Cancer	References
*Bifidobacterium*	Local delivery of bifidobacteria effectively stimulates STING signaling and increases crossover initiation of dendritic cells following anti-CD47 treatment, thereby influencing treatment	Colorectal cancer	[20]
*Enterococcus faecalis*	Promoting TH1 differentiation of CD4 + T cells and CD8 + T-cell activation	Pancreatic Cancer	[22]
*E. coli Top10*	Induction of tumor-specific CD4 + and CD8 + T cells	Colorectal cancer	[124]

#### Intratumoural microbiota: a new frontier in biotherapeutics

Recent advances in microbiology and bioengineering have inspired the development of engineered in vivo biotherapeutics for targeted cancer therapy. Large-scale sequencing is significantly expanding the range of microorganisms with potential health benefits in the context of specific diseases. Microorganisms that can participate in biotherapeutics are known as live biotherapeutics [125]. They include SCFA producers (e.g. *Enterococcus faecalis* for the treatment of inflammatory bowel disease [126]) and species of the genus Mycobacterium (e.g. *Bacteroides xylanisolvens* [127] and *Bacteroides ovatus* [128], both of which are associated with elevated levels of the TFα antigen-specific antibodies and improved immunosurveillance for cancer). Meanwhile, it is also feasible to design safe organisms or symbionts as delivery vectors of bioactive molecules or expression vectors. For example, *E.coli Nissle 1917* can be modified to combine with the surface of cancer cells and secrete a black mustard enzyme. Myrosinase is an enzyme that can convert glucosinolates into isothiocyanates, such as sulforaphane, a molecule with known anti-tumor activity. Although the biological treatment of tumors remains largely unexplored, synthetic biology holds great promise for specifically targeting tumors, actively penetrating tissues and inducing cytotoxicity in a controlled manner [129]. The therapeutic potential of *Bifidobacterium BB-12*, *Bifidobacterium Infantis strains* have been demonstrated in terms of tissue regeneration, as well as its antimicrobial and anti-inflammatory effects. Recent research has revealed that the pairing of Ecm-Alginate and *Bifidobacterium BB-12 and Bifidobacterium Infantis strains* can enhance the antibacterial and anti-inflammatory properties of the *Bifidobacterium BB-12 and Bifidobacterium Infantis strains*, thereby exhibiting some degree of anti-cancer activity. This phenomenon can be attributed to the interaction between the peptidoglycan on the extracellular polysaccharide cell membrane of the *Bifidobacterium BB-12 and Bifidobacterium Infantis strains* and Ecm-Alginate [130]. Zheng et al. observed that colonization of the TME by engineered Salmonella induced the infiltration of rich immune cells (such as monocytes/macrophages and neutrophils) through TLR4 signal transduction, and Salmonella colonized in the tumor caused the functional activation of tumor macrophages with the M1 phenotype through the secretion of Vibrio vulnificus flagellin B (FlaB) and mutual reduction in the inhibitory activity of M2-like macrophages, thereby strengthening the effect of immunotherapy [131]. The advancement of science and technology is expected to facilitate the precise and effective design and fabrication of customized biological therapeutics in synthetic biology. This development holds the potential to circumvent severe side effects caused by overdosing, thereby offering a superior solution to the challenges associated with cancer therapy (Table 10).

**Table 10:** Intratumoural microbiota that can be used in biotherapeutics

Intratumoural microbiota	Mechanism	Cancer	References
*Enterococcus faecalis,Bacteroides, xylanisolvens,Bacteroides ovatus*	Elevated levels of TFα antigen-specific antibodies	Colorectal cancer	[126–128],
*E.coli Nissle 1917*	The engineered commensal Escherichia coli bound specifically to the heparan sulphate proteoglycan on colorectal cancer cells and secreted the enzyme myrosinase to transform host-ingested glucosinolates to sulphoraphane, an organic small molecule with known anticancer activity	Colorectal cancer	[129]
*Salmonella*	1.Inducing the infiltration of abundant immune cells such as monocytes/macrophages and neutrophils via TLR4 signaling 2. The secretion of FlaB by Salmonella induces the activation of phenotype and function of tumor-associated macrophages (TAMs) with M1 phenotype, while simultaneously suppressing the M2-like inhibitory activity	Colorectal cancer, Melanoma	[131]

#### Intratumoural microbiota programming and modification: a hope for future cancer therapy

Over the past decades, chemotherapy and immunotherapy have been mainstays of cancer treatment. However, tumors have gradually developed resistance to chemotherapy drugs, and some patients who initially responded well to immunotherapy may also become resistant over time. Fortunately, with the advancement of gene editing technology, new approaches to cancer treatment, including gene engineering, have become a possibility. Compared to conventional treatment methods, gene engineering holds significant advantages as a cancer therapy [2]. Through the study of intratumoural microbiota [5], several intratumoural microbiota have been identified, including *Salmonella* [132], *E.coli* [133] and *Bifidobacterium* [134]. These bacteria can colonise and accumulate in the tumor and inhibit their growth [135]. Intratumoural microbiota colonisation provides new ideas for anti-tumor therapies. Intratumoural microbiota can be used as in vivo delivery vehicles for cancer treatment, thus overcoming the limitations of conventional anti-tumor therapies, such as toxic effects on normal tissue cells and the inability to treat deeper tumor tissues [135]. The intratumoural bacteria can therefore be modified to improve cancer treatment. For instance, Salmonella strains have been genetically manipulated to express Fas ligand (FasL) with the aim of delivering this toxic and potential antitumor cytokine to tumor sites for enhanced therapeutic efficacy. The expression of Fas ligand by Salmonella strains has been demonstrated to elicit clear antitumor responses in a Fas-dependent manner [136]. Recently, a team of researchers developed a modified strain of E. coli that effectively raised the concentration of L-arginine and enhanced T cell infiltration within the tumor microenvironment, consequently optimizing the anticancer potential [137]. While genetically engineered bacteria have exhibited remarkable potential in cancer therapy, numerous issues remain unresolved. For example, how to minimize bacterial virulence to improve safety? How to enhance the ability of bacteria to accumulate in tumor tissue? How to solve the genetic instability of genetically engineered bacteria? Overall, as medical and synthetic biology research continues to advance and more animal and human trials are conducted, genetically engineered bacterial therapy has the potential to become a significant player in future cancer treatment that cannot be overlooked (Table 11).

**Table 11:** Intratumoural microbiota that can be programmed and modified

Intratumoural microbiota	Mechanism	Cancer	References
*E. coli*	Genetically engineered E.coli increases L-arginine concentration and enhances T cell infiltration in the tumor microenvironment	Colorectal cancer	[137]
*Salmonella*	Salmonella strains were genetically modified to enable the expression of Fas ligand (FasL), which can effectively deliver the toxic but anti-tumor cytokine to the tumor site, thus enhancing the therapeutic efficacy of the treatment	Colorectal cancer Breast cancer	[136]

## Conclusion and outlook

Although the mechanisms by which microbiota colonise tumors are only just beginning to be unravelled, research is currently increasingly focused on the intratumoural microbiota. To date, each tumor is considered to be a unique bacteria composed of specific bacterial species. Even when there is a relative overlap between the primary tumor and normal tissue, intratumoural microbiota have a unique metabolic profile that is consistent with metabolites found in the associated tumor. Based on this property, intratumoural microbiota can be used as a biomarker for early screening of cancer as well as for determining prognosis, enabling timely understanding of the effectiveness of current treatment approaches and paving the way for the improvement of subsequent treatment strategies. Through appropriate programming, microbiome modulation therapies can be rationally developed, making pathogenic bacteria potentially useful as biotherapeutics and efficient intracellular anti-cancer vectors. Besides, live therapeutic bacteria have all the tools needed to rapidly deliver excellent personalised medicine, thus contributing to the development of tumor-specific therapies without serious side effects and ultimately enhancing clinical outcomes. However, due to the low biomass of intratumoural microbiota and the high risk of contamination, more research on intratumoural microbiota is currently limited to observational studies, resulting in more limited data. Therefore, future studies should closely focus on the following important aspects: (1) There are many challenges with microbial-based diagnostics, including low biomass relative to the host and confounding by reagents or environmental contaminants. Many questions regarding their uniqueness, prevalence, stability during cancer treatment, or utility during antibiotic administration remain to be answered and must be addressed prior to clinical deployment. The development of decontamination algorithms for data analysis is necessary due to the low biomass of intratumoural microbiota; (2) how intratumoural mirobiota enters and colonises the tumor; (3) culture of intratumoural mirobiota; (4) clinical translational studies of intratumoural mirobiota to foster the development of precision diagnostics and therapeutics and optimized treatment procedures for cancer patients. Furthermore, an in-depth study of different tumor microbial communities in the future may address these questions and thus resolve the existing bottlenecks, which may facilitate the modulation of intratumoural mirobiota and revolutionise anti-cancer therapeutics.

## Data Availability

Not applicable.

## Abbreviations

Abs: Antibodies
BFT: Bacteroides fragilis toxin
Cag: Cytotoxin-associated gene
CBM 588: C.butyricum MIYAIRI 588
CDT: Cytolethal distending toxin
CRC: Colorectal cancer
DCs: Dendritic cells
DSBs: Double-stranded DNA breaks
EBV: Epstein-Barr virus
ECM: Extracellular matrix
EcN: E.coli Nissle 1917
ESCC: Esophageal squamous cell carcinoma
ETBF: Enterotoxin-producing Bacteroides fragilis
FlaB: Flagellin B
Fn: Fusobacterium nucleatum
GPCRs: G Protein-Coupled Receptors
HDAC: Histone deacetylase
HPV: Human papilloma virus
LM: Listeria Monocytogenes
LTS: Long-term postoperative pancreatic cancer tumors
MAPK: Mitogen-activated protein kinase
MDSC: Myeloid-derived suppressor cells
miR: MicroRNA
NADPH: Nicotinamide adenine dinucleotide phosphate
NF-κB: Nuclear factor-κB
NO: Nitric oxide
PDA: Pancreatic ductal adenocarcinoma
pks + E.coli: Polyketidesynthase E.coli
PLC: Primary liver cancer
RFS: Recurrence-free survival
ROS: Reactive oxygen species
SCFA: Short-chain fatty acids
STING: Stimulator of interferon genes
STS: Short-term postoperative survival
TAMs: Tumor-associated macrophages
TLR-4: Toll-like receptor 4
TME: Tumormicroenvironment
TNF: Tumor necrosis factor
Treg: Regulatory T cell
VEGF: Vascular endothelial growth factor
